# Enzymes helping enzymes: Oxaloacetate decarboxylase increases malate dehydrogenase's turnover number

**DOI:** 10.1093/pnasnexus/pgaf134

**Published:** 2025-04-25

**Authors:** Gadiel Saper, Henry Hess

**Affiliations:** Department of Biomedical Engineering, Columbia University, New York, NY 10027, USA; Department of Biomedical Engineering, Columbia University, New York, NY 10027, USA

**Keywords:** enzyme kinetics, enzyme cascade, malate dehydrogenase, oxaloacetate decarboxylase

## Abstract

The catalytic performance of enzymes is largely perceived to be a property of the enzyme itself, altered by environmental conditions, such as temperature and pH. However, the maximal catalytic rates of enzymes differ up to 100-fold between in vivo and in vitro measurements, suggesting that a complex chemical system has additional effects on catalytic performance. In this work, we show that the initial rate of an enzyme can increase 3-fold due to the presence of a second enzyme, which uses the product of the first enzyme as its substrate. This enhancement may originate in an allosteric effect or result from binding competition for the product molecule by the second enzyme.

## Introduction

The field of systems chemistry aims to develop complex molecular systems showing emergent properties, such as spatial or temporal organization (1, 2). These can arise, for example, from chemically fueled motion (3), surface immobilization (4), and compartmentalized chemical networks (5). Enzymatic cascades, where the product molecules of one enzyme are the substrates for another enzyme, are one class of reaction networks capable of exhibiting complex behavior (6–8). These reaction networks form the basis of biochemical transformations, and their biotechnological use promises advances in the green production of high-value chemicals and pharmaceuticals (9). Recent studies have placed cascade enzymes in close proximity to each other using DNA or nanoparticle scaffolds, and observed an acceleration in the rate of formation of the final product (10–12). This observation was initially thought to result from the faster diffusive transfer of intermediate molecules, but kinetic models showed that this can only provide a transient boost to the throughput, and experiments with enzymes simply conjugated to each other showed no enhancement (13). An alternative explanation pointed to the effect of the scaffold on the environment of the enzyme, and experiments showed that cascade throughput can be accelerated with microenvironments pH optimized for each enzyme (13). Throughput can also increase due to the aggregation of enzymes and sequestration of intermediates by competing reactions (14). Recent experiments, however, control for the microenvironment effect and other effects by comparing the throughput of the assembled cascade on the scaffold to the individual enzymes on scaffolds and still find a several-fold, time-independent enhancement of cascade throughput (15). Thus, a mechanistic understanding of the observations is still incomplete.

Here, we show that the catalytic performance of an enzyme can be increased simply by the presence of a second enzyme that uses the product of the first enzyme as a substrate. We show that the enhancement can be explained by an increased rate of product release from the enzyme.

## Results

**Fig. 1.**
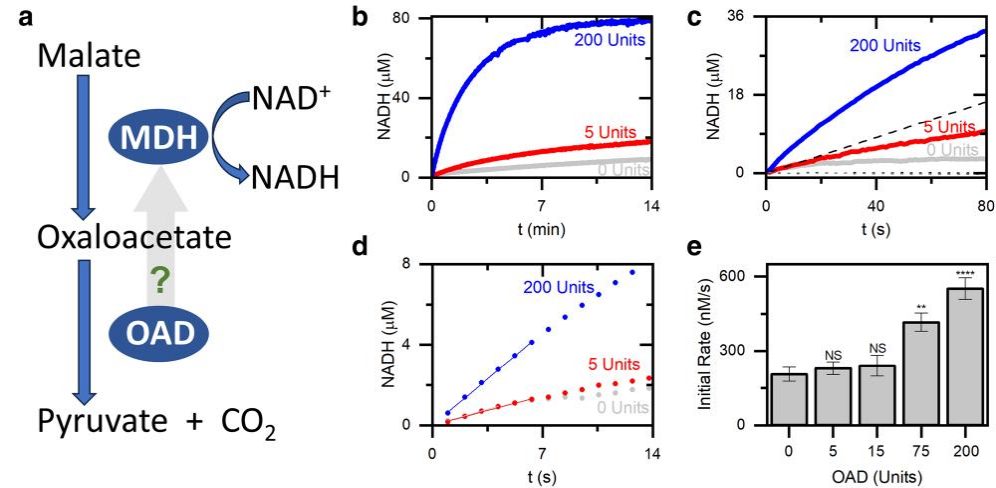
The MDH and OAD enzymatic cascade and its kinetics. a) Schematic representation illustrating the two enzymatic reactions employed in our experiment. b) Typical time-dependent NADH production by MDH in the absence of OAD (gray) and in the presence of 5 (red) and 200 (blue) units OAD per 400 µL reaction volume. c) Typical time-dependent NADH production in the first 80 s. The dashed line has the slope of the initial rate in the absence of OAD, showing what NADH production would be if it is not slowed by product inhibition and OAD does not influence MDH kinetics. The dotted black and gray lines represent control experiments with 75 units of OAD in the absence of malate or MDH, respectively. d) Zoom-in of (c); the lines depict a linear fit to determine the initial rate. e) The initial rate of NADH production for increasing OAD concentrations was averaged from eight repeated measurements for 0 units and six measurements for each of the other OAD concentrations. Error bars denote SEs. Statistical difference is calculated with a t test compared with 0 units: NS, not significant—*P* > 0.05, ****P < 0.0001, **P = 0.0016.

Our model system is the enzymatic cascade composed of malate dehydrogenase (MDH) and oxaloacetate decarboxylase (OAD; Fig. 1a), which has been previously immobilized on a DNA scaffold by Liu et al. (15). MDH converts malic acid and NAD^+^ into oxaloacetate and NADH, and OAD converts oxaloacetate into pyruvate and carbon dioxide. We measure the concentration of NADH to specifically gauge the enzymatic activity of the first enzyme in the cascade, MDH, rather than the activity of the entire enzymatic cascade. The kinetics of NADH formation by 0.5 units of MDH exhibited the anticipated behavior with an initial linear increase of the NADH concentration turning into a plateau as the equilibrium is approached (Fig. 1b). When up to 200 units of OAD were introduced, a notable increase in the reaction rate and a higher equilibrium NADH concentration were observed due to the removal of the oxaloacetate (Fig. 1b and c).

**Fig. 2.**
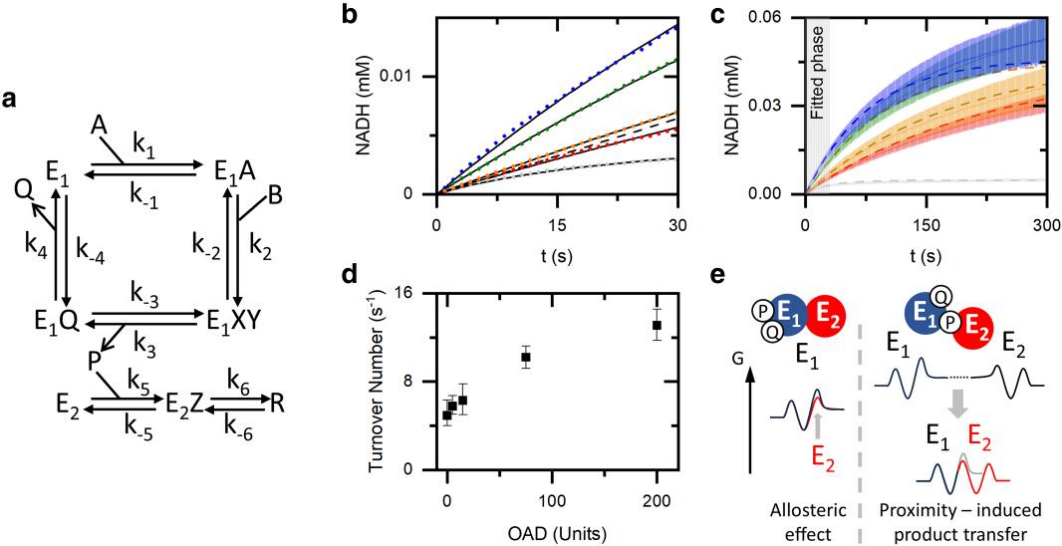
Kinetic model of the MDH-OAD cascade. a) A schematic representation of the simple ordered bi–bi kinetic path used for the fitting. Labels: *k_i_*, kinetic constants; A, malate; B, NAD; P, oxaloacetate; Q, NADH; E_1_, MDH; E_2_, OAD; R, pyruvate and CO_2_. b and c) NADH concentration (average of at least four measurements) for experiments in the absence of OAD (gray) and in the presence of 5 (red), 15 (orange), 75 (olive), and 200 (blue) units per 400 µL OAD. b) The circles are the average of the experiments, and the solid lines represent the fitted model. The dashed black line represents a theoretical model with an infinitely fast consumption of oxaloacetate but without a change in the MDH kinetic constants. c) Dashed lines represent the extrapolation of the fits to 300 s. The shaded lines represent the mean and SE of the experiments. d) The turnover number, calculated from the fitted rate constants, as a function of OAD concentration. The error bars are calculated by using the parameters obtained from fitting the upper and lower bounds of the SE seen in (c). e) A schematic representation and a sketch of the free energy along the reaction coordinate for two simplified enzymatic reactions depicting a lowering of the activation energy for conversion and product release of E_1_ by an allosteric effect of E_2_ on E_1_ and/or a proximity effect caused by the affinity of E_2_ for the product of E_1_.

We determine the initial reaction rate and find that it increases in an OAD concentration-dependent manner up to 3-fold (Fig. 1d and e). The control experiments (Fig. 1c and SI) show that this increase is not related to changes in the environment or NADH production by the OAD and its potential contaminants. Since the initial rate is not affected by product inhibition, the presence of OAD must change one or more of the rates in the MDH reaction cycle. To quantify the rate increase, we modeled the enzymatic reaction of MDH as a simple ordered bi–bi reaction following Dasika et al. (16) and the effect of the OAD as a two-step reaction reversibly converting the MDH product oxaloacetate (Fig. 2a; for full details, see SI). The fitting results show that the consumption of oxaloacetate is not sufficient to account for the increase in the initial rate, and a change in the slow steps, the product release rates *k*_3_ and *k*_4_ (16), of the MDH reaction is needed (Fig. 2b). While our fit by itself cannot reliably distinguish between changes in *k*_3_ (oxaloacetate release) and *k*_4_ (NADH release), we propose that OAD primarily affects oxaloacetate release. Adjusting only *k*_3_ and *k*_−3_ is sufficient to fit the experimental results and explain the increase in the initial rate (Fig. 2b and c).

The turnover number, $\tau^{-1}$, was calculated from the kinetic model parameters for each experimental condition as


(1) $$\tau^{-1}={\left(\frac{1}{k_{3}}+\frac{1}{k_{4}}\right)}^{-1}$$

where *τ* is the time required to produce one NADH at saturating substrate concentrations. The increase in the calculated turnover numbers with increasing OAD concentrations (Fig. 2d) mirrors the increase in the initial reaction rates (Fig. 1e) but is based on the fitting of a longer time period, confirming that the observed rate enhancement upon OAD addition can only result from an increase in the forward reaction rates. Given the known affinity between OAD and its substrate oxaloacetate, we hypothesize that the observed increase in the initial reaction rate is primarily due to an increase in *k*_3_, the rate constant associated with the release of oxaloacetate from MDH.

## Discussion

A 3-fold acceleration of the conversion and product release step of MDH by interacting with OAD implies a lowering of the activation energy by one *k*_B_*T*. This may be the result of an allosteric effect caused by direct binding of OAD to MDH or competition for the product molecule between MDH and OAD, or a combination of both (Fig. 2e). While further research into a mechanistic explanation of the effect is needed, it is clear that OAD directly accelerates MDH catalysis. The observed large acceleration of one enzyme in a cascade by the presence of a downstream enzyme may be frequently—although not always—present when enzymes are in proximity and can suitably interact. This highlights that the catalytic performance of an enzyme in vivo is an emergent phenomenon depending not only on substrate concentrations, solvent properties, and the presence of cofactors and inhibitors, but also on the presence of other molecular machines at high local concentrations.

## Materials and methods

The experimental measurements were conducted using a spectrophotometer, with a final reaction volume of 400 µL in HEPES buffer (pH 7.2) with 5 mM MnCl_2_, 2 mM NAD^+^, 2 mM malic acid, 0.5 units (93 nM) MDH (units are measured for the reverse reaction), and OAD concentrations ranging from 0 to 200 units (0–1,564 nM). NADH concentrations were quantified via absorption measurements at 340 nm. Kinetic modeling was performed using KinTek Explorer (17). See SI for more details.

## Supplementary Material

pgaf134_Supplementary_Data

## Data Availability

All data are presented in the manuscript. Raw data are available at: https://doi.org/10.7916/e015-zq67.
