# Supplementary material for: Enzymes helping enzymes: Oxaloacetate decarboxylase increases malate dehydrogenase's turnover number
Source: PNAS Nexus. 2025 Apr 25;4(5):pgaf134. doi: 10.1093/pnasnexus/pgaf134 (PMC12048710; doi:10.1093/pnasnexus/pgaf134)
Supplement: pgaf134_Supplementary_Data [file pgaf134_supplementary_data.pdf]

PNAS nexus

Supplementary Information for
Enzymes Helping Enzymes: Oxaloacetate Decarboxylase Increases Malate Dehydrogenase's Turnover Number

Gadiel Saper, Henry Hess

Department of Biomedical Engineering, Columbia University, New York, NY, USA

* Henry Hess

Email: hh2374@columbia.edu

This PDF file includes:

Supplementary text

Supplementary Information Text

Expanded Method:

Reagents: NAD+ (Sigma, NAD Grade I, free acid) and Malic Acid (Sigma, L-(-)-Malic acid, ReagentPlus®, ≥99%) were separately dissolved in 100 mM HEPES Buffer, pH 7.2, to attain final concentrations of 20 mM and 100 mM, respectively. These solutions were utilized within 4 hours of preparation. Lyophilized MDH (Creative Enzymes, Native Microorganism Malate Dehydrogenase), was stored at -20 °C and reconstituted in 100 mM HEPES Buffer, pH 7.2. It was kept on ice and used within 3 hours of preparation. The Michaelis Constants provided by the supplier for this MDH are 5.4×10^{-5} M (L-Malate), 5.0×10^{-6} M (Oxaloacetate), and 8.1×10^{-6} M (NADH). Lyophilized OAD (Sigma, Oxaloacetate Decarboxylase from Pseudomonas) was stored at -20 °C, reconstituted in 100 mM HEPES Buffer, pH 7.2, it was kept on ice and used within 3 hours of preparation. Citrate Synthase (Creative Enzymes, Recombinant Porcine Citrate Synthase) was stored at -80 °C and diluted in 100 mM HEPES Buffer, pH 7.2, kept on ice and used within 3 hours of preparation. Acetyl coenzyme A (Sigma, Acetyl coenzyme A trisodium salt) was stored at -20 °C and reconstituted in 100 mM HEPES Buffer, pH 7.2. It was kept on ice and used within 3 hours of preparation.

Measurements: The experimental solution comprised 2 mM NAD^+, 2 mM Malic Acid, 5 mM $MnCl_2$, 0.5 units (0.093 μM) of MDH (units provided for the reverse reaction), and between 0 and 200 units (0 – 1.564 μM) of OAD (measured by the supplier), all in 100 mM HEPES Buffer, pH 7.2, to achieve a final volume of 400 µl. Control measurements were conducted by substituting enzymes and/or substrates with an equivalent volume of 100 mM HEPES Buffer, pH 7.2. All experiments were monitored at 340 nm using a spectrophotometer (Evolution 201, Thermo Scientific). The temperature during the experiments was 19 ± 1 °C. For each measurement, the blank was measured before adding the malic acid and for clarity the background was also adjusted by fitting the first few points to a linear curve and using the intercept at t = 0 (defined as the time point when malic acid was added) as the background absorbance. The NADH concentration was calculated utilizing a molar absorptivity of $\varepsilon_{340} = 6220\ M^{-1}cm^{-1}$.

To calculate the initial rate a linear curve was fit to the first few seconds of each measurement. To further evaluate the initial rate, we performed measurements with a lower concentration of OAD (5 and 15 units) where the initial rate persists longer due to the consumption of Oxaloacetate. No significant increase in the initial rate compared to experiments without OAD was found.

To control for any potential non-specific effects of the OAD solution, a heat-inactivated control was prepared. OAD was inactivated by incubation at 100 °C for 30 minutes followed by immediate cooling on ice for 30 minutes. The initial rate of NADH production in the presence of 75 Units of heat-inactivated OAD (120 ± 18 $nM\cdot s^{-1}$) was not significantly different from the rate observed in the absence of OAD, confirming the specificity of the observed effects to active OAD.

To test if the effect on MDH is specific to OAD, 0.6 mM Acetyl coenzyme A was added and 0 – 3000 units (0 – 1.04 μM) of Citrate Synthase was used instead of OAD. The Citrate Synthase units were measured by the supplier and are defined as the amount of enzyme that will generate 1.0 nmol of TNB per minute at 37 ºC. Measurements with 400 units of Citrate Synthase and no MDH or no Malate did not show NADH production. The initial rates measured were: 0U: 175 ± 30, 5U: 141 ± 4, 100U: 195 ± 44, 200U: 266 ± 40, 400U: 275 ± 72, 3000U: 518 ± 42 $nM\cdot s^{-1}$ (average ± standard error). The temperature for these measurements was 19 °C, except for the 3000U data point which was measured at 22 °C (causing a 35% acceleration relative to a measurement at 19 °C based on the manufacturer's data). Only the temperature-corrected 3000U measurement (384 ± 31 $nM\cdot s^{-1}$) was significantly different from the 0U measurement with p = 0.02. Although the OAD units and the Citrate Synthase units are defined differently and cannot be

compared directly, the enzyme concentrations can be calculated and compared. Similar concentrations of OAD and Citrate Synthase appear to have similar effects on the MDH activity, but additional measurements are required to fully characterize the effect.

Modeling: Kinetic modeling and data fitting were performed using KinTek Explorer software version 11.1.1 (KinTek Corporation). The following initial concentrations were used for the fitting: 2 mM NADH, 2 mM Malic Acid, 50 nM MDH, and 0, 39, 117, 587, and 1564 nM for 0, 5, 15, 75 ,200 Units of OAD respectively. The data was fitted by regression analysis to produce a minimum chi^2.

To determine the kinetic parameters of the MDH reaction, we initially fit the data obtained in the absence of OAD (average of 7 measurements). We focused on the first 30 seconds of the reaction, where the initial rate is most prominent. Utilizing the parameters obtained by Dasika et al. (1) as an initial guess, we fit the data to a bi-bi kinetic model, first leaving all rate constants as free parameters to obtain initial estimates for all kinetic parameters. Subsequently, each parameter was varied individually to refine its estimate and determine its standard error. This iterative fitting process yielded the following parameters: k_1 = 12500 ± 400 $(mM \cdot s)^{-1}$, k_{-1} = 46900 ± 1700 s^{-1}, k_2 = 7590 ± 220 $(mM \cdot s)^{-1}$, k_{-2} = 678 ± 21 s^{-1}, k_3 = 5.49 ± 0.04 s^{-1}, k_{-3} = 24900 ± 400 $(mM \cdot s)^{-1}$, k_4 = 47.4 ± 0.6 s^{-1}, k_{-4} = 5620 ± 280 $(mM \cdot s)^{-1}$. These rates correspond to an equilibrium constant of $K_{eq} = \frac{K_1 K_2 K_3 K_4}{K_{-1} K_{-2} K_{-3} K_{-4}} = 6 \cdot 10^{-6}$, which is close to the value of $5 \cdot 10^{-6}$ at pH 7 given in supplementary reference (1).

To obtain the kinetic constants for the OAD enzyme we used the measurements with 5U of OAD. At this concentration, the initial rate of NADH production remains largely unchanged compared to the 0U measurement, but a change is observed at longer time frames, suggesting that the parameters for MDH should remain mostly unchanged. We fit the first 300 s by leaving all OAD rate constants as free parameters to obtain initial estimates for all kinetic parameters. Subsequently, each parameter was varied individually to refine its estimate and determine its standard error. The rates obtained are: k_5 = 15600 ± 30 $(mM \cdot s)^{-1}$, k_{-5} = 1460 ± 10 s^{-1}, k_6 = 2560 ± 10 s^{-1}, k_{-6} = 278 ± 2 s^{-1}.

The data obtained with all OAD concentrations (5, 15, 75, 200 units average of 6, 6, 6, 4 measurements respectively) were fit for the first 30 s with the above set of fixed parameters, with the exception of the rate constant for oxaloacetate release from MDH (k_3), which was allowed to vary. k_{-3} was adjusted to maintain the equilibrium constant. The rates obtained from the fitting are: 5U: k_3 = 6.58 ± 0.04 s^{-1}, k_{-3} = 29000 $(mM \cdot s)^{-1}$, 15U: k_3 = 7.26 ± 0.01 s^{-1}, k_{-3} = 33000 $(mM \cdot s)^{-1}$, 75U: k_3 = 13.00 ± 0.03 s^{-1}, k_{-3} = 59000 $(mM \cdot s)^{-1}$, 200U: k_3 = 18.1 ± 0.1 s^{-1}, k_{-3} = 82000 $(mM \cdot s)^{-1}$.

For the curve in Figure 2 showing the NADH production if the parameters for MDH are maintained but oxaloacetate is immediately removed from the system (infinitely fast consumption of oxaloacetate), a 1 M OAD concentration, and rates of $k_5 = 10^8$ $(mM \cdot s)^{-1}$, k_{-5} = 0 s^{-1}, $k_6 = 10^8$ s^{-1}, k_{-6} = 0 s^{-1} were used in the simulation. All other rates were the same as in the 0 U OAD fit.

To calculate the error of the turnover number we fit the k_3 for the average plus and minus the standard error for each OAD concentration and used that to calculate the upper and lower limit of the turnover number.

References:

(1) Dasika SK, Vinnakota KC, Beard DA. 2015. Determination of the catalytic mechanism for mitochondrial malate dehydrogenase. *Biophys. J.* 108, 408–419.
